# First evidence on ergonomic exposures and musculoskeletal pain among the Faroese workforce

**DOI:** 10.1007/s00420-026-02221-7

**Published:** 2026-07-06

**Authors:** Rúni Bláfoss, Lars Louis Andersen, Simone Vestergaard Christiansen, Thomas Clausen, Pál Weihe, Annika Helgadóttir Davidsen

**Affiliations:** 1 National Research Centre for the Working Environment, Lersø Parkalle 105, DK-2100 Copenhagen, Denmark; 2 Department of Research, National Hospital of the Faroe Islands, Tórshavn, Faroe Islands; 3 Faculty of Health, University of the Faroe Islands, Tórshavn, Faroe Islands

**Keywords:** Working conditions, Musculoskeletal diseases, Low back pain, Neck pain, Shoulder pain, Epidemiology

## Abstract

**Objectives:**

The Faroe Islands has historically been a fishing society, with workers being exposed to high physical work demands. However, the work environment has never been systematically studied. This study investigates associations of ergonomic exposures at work with low-back pain (LBP) and neck/shoulder pain (NSP) intensity.

**Methods:**

This cross-sectional questionnaire survey included 5198 workers (∼24% of the country’s workforce), with > 3800 participants in the main analyses (∼18% of the country’s workforce). General linear models tested associations of specific ergonomic exposures and a combined ergonomic index with LBP and NSP intensity, respectively, while adjusting for relevant confounders.

**Results:**

Mean LBP and NSP intensity were 2.6 (± 2.8) and 3.0 (± 2.7) points, respectively, on a 0–10 numeric rating scale. All ergonomic exposures showed strong exposure-response associations with LBP and NSP intensity, with the exception of ‘standing/walking’, which showed no significant association with NSP intensity. The strongest associations for both women and men were observed for ‘pushing/pulling’ and ‘carrying/lifting’ with LBP intensity, and ‘back bent/twisted’ and ‘pushing/pulling’ with NSP intensity. The ergonomic index (scale 0-100) showed clear exposure-response associations; women and men in the highest ergonomic demand category (> 40) reported 2.41 and 1.42 points higher LBP intensity, respectively, while all workers showed 1.51 points higher NSP intensity.

**Conclusions:**

This first evidence of the ergonomic working environment in the Faroe Islands demonstrates a general working population with relatively intense pain and clear exposure-response associations between level of ergonomic demands and higher musculoskeletal pain intensity – for both women and men. Mapping these associations among the general working population in the Faroe Islands provides essential knowledge for future occupational health initiatives.

**Supplementary Information:**

The online version contains supplementary material available at 10.1007/s00420-026-02221-7.

## Introduction

The Faroe Islands, an autonomous territory within the Kingdom of Denmark, consists of 18 islands and approximately 55,000 inhabitants in the North Atlantic Ocean between Great Britain and Iceland. Historically reliant on fishing and agriculture, the workforce faced high physical work demands. While the country has evolved into a modern society with increasingly diverse service industries, and physical work demands have likely decreased on a societal level in recent decades, no systematic mapping of the general Faroese workforce has been conducted to date.

As a small, distinct society, the Faroe Islands presents a unique setting for nationwide occupational health initiatives, providing a valuable context for evaluating interventions to reduce physical work demands and musculoskeletal pain. The small population and potential for high intervention adherence make empirical knowledge gained from this context particularly valuable for translation and scaling to larger populations.

Previous research demonstrates that physically demanding work increases the risk of developing musculoskeletal pain (da Costa and Vieira 2010; Boschman et al. 2011; Coenen et al. 2014a, b; Andersen et al. 2021a, b; Kuijer et al. 2024; Oakman et al. 2025; Øverås et al. 2020). High physical work demands include activities such as lifting and carrying, bending and twisting the back, and working with arms elevated above shoulder height (da Costa and Vieira 2010; Andersen et al. 2021a, b; Kuijer et al. 2024), and such occupational exposures are associated risk of musculoskeletal pain, short- and long-term sickness absence, and disability pension (da Costa and Vieira 2010; Coenen, Gouttebarge, et al. 2014; Andersen et al. 2021a, b; Kuijer et al. 2024; Sterud 2014; Sundstrup et al. 2017; d’Errico et al. 2020; Øverås et al. 2020; Thorsen et al. 2021; Bláfoss et al. 2021; Pedersen et al. 2022; Skovlund et al. 2023; Oakman et al. 2025).

Workers in physically demanding occupations are typically exposed to multiple ergonomic risk factors throughout their working day, including prolonged standing/walking, arm elevation, and squatting/kneeling, which may cumulatively increase physical work demands and musculoskeletal pain (Andersen et al. 2021a, b; Andersen et al. 2021a, b). Complete assessment of ergonomic work demands therefore requires investigating not only individual ergonomic exposures but also their combined effects across all exposure levels and their associations with pain intensities.

Musculoskeletal pain intensity carries serious long-term consequences. Recent prospective cohort studies with register follow-up have identified dose-response associations between higher pain intensity and increased risk of long-term sickness absence and disability pension among both the general working population in Denmark and eldercare workers (Skovlund et al. 2023; Bláfoss et al. 2021). These findings suggest that musculoskeletal pain intensity serves as an important predictor of severe outcomes, including work absenteeism and permanent work disability.

Notably, musculoskeletal pain is complex and may originate from interacting biological, psychological, and social factors (Engel 1977). Work exposures are therefore not limited to biomechanical or ergonomic demands. Psychosocial conditions at work may also influence the development and experience of musculoskeletal pain (Bongers et al. 1993; Hauke et al. 2011; Lang et al. 2012; Oakman et al. 2025) and may confound associations between ergonomic exposures and pain because they may coexist with physical work demands. Influence at work and emotional demands may represent two psychosocial factors associated with pain. While low influence at work may restrict workers’ possibilities to plan work tasks, regulate work pace, vary postures and movements, and take short recovery breaks during the working day, high emotional demands may contribute to stress-related physiological responses, particularly frequent in jobs involving intensive contact with clients, patients, pupils, or other service users (Arbejdstilsynet 2023). Previous reviews and meta-analyses have shown that psychosocial work factors, including high demands and low influence/autonomy, are associated with musculoskeletal disorders and pain in the low back, neck, and shoulders (Bongers et al. 1993; Hauke et al. 2011; Lang et al. 2012; Oakman et al. 2017, 2025). Thus, considering psychosocial factors is crucial when examining associations between ergonomic demands and pain.

This holistic view that the working environment comprises both physical and psychosocial factors elaborates on the recent discussion paper by Holtermann and colleagues about the newly developed CoWork musculoskeletal health model, suggesting a paradigm shift in the work environment research from risk reductions to health promotion (Holtermann et al. 2026). The authors argue that both physical and psychosocial work factors may not only represent risk factors for poor health, but they may also serve as work factors promoting musculoskeletal health.

However, given that evidence-based knowledge is essential for developing and implementing effective workplace health initiatives, addressing knowledge gaps among the Faroese workforce is crucial. As this study represents the first broad mapping of ergonomic working conditions and musculoskeletal pain in the general working population of the Faroe Islands, we included all available ergonomic exposure variables from the questionnaire to describe the overall pattern of ergonomic exposures and their associations with pain intensity in body regions assessed in the survey. The present study therefore aims to investigate associations between specific ergonomic work demands and cumulative ergonomic exposure with low-back pain (LBP) and neck/shoulder pain (NSP) intensity. We hypothesized that all ergonomic factors as well as cumulative ergonomic demands would associate in an exposure-response fashion with higher pain intensities.

## Methods

### Study design and population

This cross-sectional study stems from the first nationwide questionnaire survey mapping the working environment among the general working population in the Faroe Islands. Data collection spanned from April to December 2021. Eligible participants were ≥ 18 years old, speaking and/or understanding Faroese, and currently employed in a Faroese workplace.

The survey was administered online. Recruitment occurred in three stages: initially through radio, TV, and social media; subsequently via major employers; and finally through invitations sent to all workers via digital post and postcards to all households (Christiansen et al. 2024).

In April 2021, the Faroese workforce comprised approximately 22,000 individuals (Hagstovan 2025a). Of these, 9420 completed or partially completed the questionnaire (∼43%). The total sample in the present study comprised 5198 participants who responded to essential questions on ergonomic exposures and pain intensity, while 3853 were included in the main analyses (∼18% of the total workforce). The study conforms to the STROBE guidelines (von Elm et al. 2008).

## Ergonomic exposures (exposure)

The following questions assessed the ergonomic exposures: ‘*How large part of your working time do you* 1) sit, 2) walk or stand, 3) work with your back twisted or bent forward without being able to support with your hands and arms, 4) lift your arms at or above shoulder height, 5) do repetitive arm movements several times per minute (e.g. packing work, assembly, or cutting tasks, 6) squat or kneel, 7) push or pull, and 8) carry or lift’. The response options were: ‘1) Never, 2) Seldom, 3) Approximately ¼ of the time, 4) Approximately ½ of the time, 5) Approximately ¾ of the time, and 6) Almost all the time’. Response categories were converted to 0%, 12.5%, 25%, 50%, 75%, and 100% of working time. Mean exposure values were then calculated to create an ergonomic index of 0-100, categorized as low (0–10), moderate (> 10–20), somewhat high (> 20–30), high (> 30–40), and very high (> 40) ergonomic demands (Andersen et al. 2021a, b).

All seven ergonomic exposure variables available from the questionnaire were included to support the study’s broad mapping aim and to allow comparison of exposure-pain patterns across the body regions assessed.

## Musculoskeletal pain (outcome)

Musculoskeletal pain intensity was assessed with the validated numeric rating scale (NRS) from 0 to 10 with the questions *‘How much pain have you had in the lower back the past 3 months’* and *‘How much pain have you had in your neck or shoulders the past 3 months’*, with 0 representing “no pain at all” and 10 representing “worst imaginable pain” (Downie et al. 1978).

## Ethical aspects

According to Faroese and Danish legislations, ethical approval and informed consent are not required for scientific studies employing anonymized or pseudo-anonymized questionnaires. All data were securely stored on a secure drive and fully de-identified and anonymized prior to statistical analysis.

## Control variables

Analyses were adjusted for relevant covariates. The minimally adjusted model controlled for age (continuous) and sex (categorical: male/female) due to their association with pain (Hoy et al. 2012; Hartvigsen et al. 2018). Subsequently, we stepwise adjusted for education (categorical), work-related psychosocial factors – influence at work (continuous) and emotional demands (continuous) – from the validated ‘Danish Psychosocial Work Environment Questionnaire (DPQ)’ (Clausen et al. 2019), and diagnosed osteoarthritis (categorical: yes/no). Response options for influence at work were *‘1) To a very small extent*,* 2) to a small extent*,* 3) somewhat*,* 4) to a large extent*,* and 5) to a very large extent’*. Response options for emotional demands at work were *‘1) Never/almost never*,* 2) seldom*,* 3) sometimes*,* 4) often*,* and 5) always’.* Responses about influence at work and emotional demands were converted to 0, 25, 50, 75, and 100, respectively. Influence at work comprised five items and emotional demands four. We used the mean composite scores for further analysis.

Education was included as a proxy for unmeasured lifestyle factors as weight, smoking status, and leisure-time physical activity (Danish Health Authority 2022). Influence at work and emotional demands were included due to their associations with the development of musculoskeletal pain, including LBP and NSP (Bongers et al. 1993; Hauke et al. 2011; Lang et al. 2012; Clausen et al. 2013; Oakman et al. 2017, 2025). These two factors from the DPQ were chosen a priori to represent one central psychosocial resource (influence/autonomy) and one central psychosocial demand (emotional demands). This choice also reflects the demand-control perspective, in which influence/autonomy is considered important for work-related strain (Karasek 1979), while emotional demands capture an important stressor in contemporary service- and care-oriented work. Finally, osteoarthritis functions as a confounder affecting both exposure and outcome.

### Statistical analyses

All analyses were performed in R (version 4.5.1) using RStudio (version 2025.05.1–513). Associations between ergonomic exposures (exposures) and pain intensity (outcomes) were tested using general linear models (glm function) while controlling for confounders. Interaction analyses were performed to examine interactions with sex. Because sex interacted for LBP, but not NSP, stratified analyses for sex were performed for all associations between ergonomic exposures and LBP. Trend tests tested exposure-response associations. Sensitivity analyses excluded (1) workers with serious illness (e.g. cancer, myocardial infarction, ischemic stroke), (2) workers working between 20 and 60 h/week, and (3) workers working between 35 and 45 h/week. Furthermore, we calculated the Spearman correlations between all seven ergonomic exposures. Descriptive statistics of pain intensity across the Faroese International Standard Classification of Occupations (FISCO) were also performed. Because FISCO-group 2 (‘Professionals’) comprised heterogeneous subgroups – professionals, healthcare workers, and teachers/childcare workers – and group 5 (‘Service and Sales Workers’) included care workers, we reclassified these categories into an expanded FISCO-11 code. Here, group 2 was divided into its three subgroups, and care workers were reassigned to the healthcare sector, following the original protocol (Christiansen et al. 2024). An alpha level of < 0.05 was considered statistically significant, and estimates are reported as beta-coefficients between the exposure levels compared to the reference group in pain intensity (NRS 0–10) with 95% confidence intervals (CI).

## Results

### Participant characteristics

Table 1 shows that more women than men participated in the study, and that the largest FISCO-group was ‘2: Professionals’, while the smallest was ‘6: Skilled Agriculture, Forestry, and Fishing Workers’. The included workers show higher NSP intensity than LBP intensity.

**Table 1:** Participant characteristics

Participant characteristics	*N*	%	Mean	SD
Age	5198		46.5	12.8
Sex	5197			
Women	3121	60.1		
Men	2076	39.9		
FISCO				
1: Managers	451	8.7		
2: Professionals	1992	38.3		
3: Technicians and associate professionals	760	14.6		
4: Clerical support workers	664	12.8		
5: Service and sales workers	557	10.7		
6: Skilled agriculture, forestry and fishing workers	71	1.4		
7: Craft and related trades workers	247	4.8		
8: Plant and machine operators, and assemblers	195	3.8		
9: Elementary occupations	246	4.7		
Low-back pain intensity past three months (NRS 0–10)	3830		2.6	2.8
Neck/shoulder pain intensity past three months (NRS 0–10)	3853		3.0	2.7

FISCO, Faroese international standard classification of occupations (ISCO-08); NRS, numeric rating scale; N, number of participants; SD, standard deviation

**Table 2:** Descriptive statistics of low-back pain (LBP) and neck/shoulder pain (NSP) intensity, respectively, across workers from the original nine FISCO-groups (upper part of table) and the revised 11 FISCO-groups (lower part of table) included in the regression analyses

FISCO	*N*	LBP (95% CI)	*N*	NSP (95% CI)
1: Managers	334	1.84 (1.58–2.11)	333	2.35 (2.09–2.60)
2: Professionals	1499	2.59 (2.44–2.73)	1505	2.94 (2.81–3.08)
3: Technicians and associate professionals	590	2.33 (2.12–2.53)	602	2.71 (2.51–2.91)
4: Clerical support workers	490	2.28 (2.04–2.52)	503	3.26 (3.02–3.51)
5: Service and sales workers	381	3.31 (3.00-3.61)	380	3.48 (3.19–3.76)
6: Skilled agriculture, forestry, and fishing workers	53	2.42 (1.70–3.13)	54	2.65 (1.99–3.30)
7: Craft and related trades workers	171	2.49 (2.11–2.88)	167	2.90 (2.50–3.29)
8: Plant and machine operators, and assemblers	131	3.63 (3.12–4.15)	131	4.14 (3.63–4.64)
9: Elementary occupations	173	3.84 (3.38–4.30)	170	3.97 (3.54–4.40)

FISCO-11	N	LBP (95% CI)	N	NSP (95% CI)
1: Managers	334	1.84 (1.58–2.11)	333	2.35 (2.09–2.60)
2: Professionals	510	1.88 (1.66–2.09)	510	2.34 (2.13–2.56)
3: Health care sector workers	470	3.27 (3.01–3.54)	469	3.44 (3.19–3.69)
4: Teachers and childcare workers	671	2.95 (2.72–3.18)	675	3.33 (3.12–3.55)
5: Technicians and associate professionals	590	2.33 (2.12–2.53)	602	2.71 (2.51–2.91)
6: Clerical support workers	490	2.28 (2.04–2.52)	503	3.26 (3.02–3.51)
7: Service and sales workers	229	2.90 (2.52–3.29)	231	3.01 (2.66–3.36)
8: Skilled agriculture, forestry, and fishing workers	53	2.42 (1.70–3.13)	54	2.65 (1.99–3.30)
9: Craft and related trades workers	171	2.49 (2.11–2.88)	167	2.90 (2.50–3.29)
10: Plant and machine operators, and assemblers	131	3.63 (3.12–4.15)	131	4.14 (3.63–4.64)
11: Elementary occupations	173	3.84 (3.38–4.30)	170	3.97 (3.54–4.40)

Pain estimates are presented as mean pain intensity and 95% confidence intervals (95% CI). See ‘Statistical analyses’ for specifications about the two FISCO-analysesFISCO, Faroese international standard classification of occupations; N, number of participants; LBP, low-back pain; NSP, neck/shoulder pain; CI: confidence intervals

### FISCO-groups and musculoskeletal pain

Table 2 illustrates LBP and NSP intensities across the original FISCO-groups (upper part of table) and the revised FISCO-11 (lower part of table) (see ‘Statistical analyses’). In the original FISCO, group 1 showed the lowest pain intensity, while groups 8 and 9 (10 and 11 in FISCO-11) showed the highest. In FISCO-11, groups 1 and 2 showed lowest pain intensities with almost identical estimates. FISCO-11 revealed substantial differences among groups 2–4: ‘2: Professionals’ reported markedly lower pain intensities than ‘3: Health Care Sector Workers’ and ‘4: Teachers and Childcare Workers’. Pain levels in ’7: Service and Sales Workers’ is lower in FISCO-11 compared to the original FISCO, reflecting the relocation of the ‘Care work’ subgroup (with higher pain) to the ‘3: Health Care Sector Workers’. This subgroup analysis indicates that the original FISCO-group 2 encompassed occupations with substantial within-group variation in pain levels. In FISCO-11, ‘2: Professionals’ reported LBP and NSP intensities of 1.88 (95% CI 1.66–2.09) and 2.34 (95% CI 2.13–2.56), respectively, while ‘3: Health Care Sector Workers’ and ‘4: Teachers and Childcare Workers’ reported mean LBP intensities of 3.27 (95% CI 3.01–3.54) and 2.95 (95% CI 2.72–3.18), respectively. The corresponding mean NSP intensities were 3.44 (95% CI 3.19–3.69) and 3.33 (95% CI 3.12–3.55), respectively.

### Specific exposures and musculoskeletal pain

#### Low-back pain (LBP)

Table 3 shows that 2280 women were included in this analysis. For ‘standing/walking’, an association existed between standing/walking for 75% and 100% of the workday and higher LBP intensity in model 1, while standing/walking for 100% of the workday reached significance in model 2 and 3. All other ergonomic exposures showed statistically significant associations from 12.5% and up to 100% of the workday. Trend tests showed very strong exposure-response associations between ergonomic exposures and LBP intensity among women (trend test: *p* < 0.0001).

Table 4 shows that 1550 men were included in the analysis. ‘Standing/walking’ for 75% and 100% of the workday was associated with higher LBP intensity in model 1, while no associations were observed in models 2 and 3. All other ergonomic exposures showed statistically significant associations from 12.5% and up to 100% of the workday, except for ‘back bent/twisted’ for 12.5% of the workday in model 1, and for ‘squatting/kneeling’ for 75% of the workday in models 2 and 3. The* p*-value for the trend test for ‘standing/walking was* p* < 0.05, while all other ergonomic exposures showed very strong exposure-response associations (* p* < 0.001).

In a separate adjusted analysis, women had slightly higher LBP intensity than men after adjustment for age, education, influence at work, emotional demands, and osteoarthritis (adjusted mean difference 0.33 points (95% CI 0.15 to 0.52, *p* < 0.001)).

**Table 3:** Associations between level of specific ergonomic exposures and differences in low-back pain (LBP) intensity compared to no exposure among *women*, presented as difference in LBP intensity and 95% confidence intervals

% of work time	*N*	%	Model 1	Model 2	Model 3
Standing/walking	2280				
0	25	1.1	0	0	0
12.5	594	26.1	0.26 (− 0.87-1.40)	0.35 (− 0.78-1.47)	0.51 (− 0.59-1.61)
25	382	16.8	0.61 (− 0.53-1.75)	0.65 (− 0.49-1.79)	0.64 (− 0.47-1.75)
50	408	17.9	1.03 (− 0.11-2.17)	1.01 (− 0.13-2.15)	0.76 (− 0.35-1.87)
75	344	15.1	**1.17 (0.03–2.32)**	1.14 (− 0.01-2.28)	0.78 (− 0.34-1.90)
100	527	23.1	**2.15 (1.02–3.28)**	**1.97 (0.84–3.10)**	**1.60 (0.50–2.71)**
Back bent/twisted	2280				
0	950	41.7	0	0	0
12.5	685	30.0	**0.76 (0.49–1.02)**	**0.65 (0.38–0.92)**	**0.49 (0.22–0.76)**
25	247	10.8	**1.83 (1.45–2.21)**	**1.68 (1.29–2.07)**	**1.37 (0.98–1.77)**
50	165	7.2	**2.35 (1.89–2.80)**	**2.18 (1.72–2.64)**	**1.85 (1.39–2.31)**
75	113	5.0	**2.90 (2.37–3.44)**	**2.74 (2.20–3.28)**	**2.41 (1.87–2.95)**
100	120	5.3	**2.83 (2.31–3.35)**	**2.57 (2.03–3.10)**	**2.18 (1.65–2.71)**
Arms elevated	2280				
0	907	40.0	0	0	0
12.5	912	40.0	**0.96 (0.70–1.22)**	**0.83 (0.57–1.10)**	**0.55 (0.29–0.82)**
25	249	10.9	**1.89 (1.49–2.28)**	**1.66 (1.26–2.07)**	**1.30 (0.89–1.70)**
50	110	4.8	**2.09 (1.54–2.65)**	**1.88 (1.32–2.44)**	**1.37 (0.82–1.93)**
75	61	2.7	**2.26 (1.53–2.99)**	**2.00 (1.27–2.73)**	**1.61 (0.88–2.33)**
100	41	1.8	**2.31 (1.43–3.19)**	**1.99 (1.10–2.87)**	**1.49 (0.62–2.37)**
Repetitive work	2280				
0	1541	67.6	0	0	0
12.5	406	17.8	**0.67 (0.37–0.98)**	**0.58 (0.27–0.89)**	**0.42 (0.11–0.72)**
25	111	4.9	**1.85 (1.30–2.39)**	**1.65 (1.10–2.20)**	**1.43 (0.90–1.97)**
50	68	3.0	**2.13 (1.44–2.82)**	**1.97 (1.28–2.66)**	**1.82 (1.15–2.49)**
75	55	2.4	**1.85 (1.09–2.61)**	**1.58 (0.81–2.35)**	**1.37 (0.63–2.12)**
100	99	4.3	**1.84 (1.26–2.41)**	**1.55 (0.96–2.13)**	**1.42 (0.85–1.99)**
Squatting/kneeling	2280				
0	1273	55.8	0	0	0
12.5	589	25.8	**1.01 (0.74–1.28)**	**0.89 (0.61–1.17)**	**0.67 (0.39–0.95)**
25	260	11.4	**2.03 (1.66–2.41)**	**1.84 (1.46–2.22)**	**1.47 (1.08–1.85)**
50	92	4.0	**2.63 (2.04–3.22)**	**2.43 (1.83–3.02)**	**2.04 (1.44–2.63)**
75	40	1.8	**1.91 (1.03–2.78)**	**1.68 (0.80–2.55**	**1.40 (0.54–2.26)**
100	26	1.1	**2.73 (1.64–3.81)**	**2.58 (1.50–3.66)**	**2.30 (1.23–3.38)**
Pushing/pulling	2280				
0	1342	58.9	0	0	0
12.5	570	25.0	**0.88 (0.60–1.15)**	**0.75 (0.48–1.03)**	**0.57 (0.29–0.84)**
25	204	9.0	**1.88 (1.47–2.29)**	**1.72 (1.30–2.14)**	**1.40 (0.98–1.81)**
50	81	3.6	**2.68 (2.05–3.30)**	**2.45 (1.82–3.08)**	**2.00 (1.38–2.63)**
75	43	1.9	**3.13 (2.28–3.97)**	**2.83 (1.98–3.67)**	**2.39 (1.55–3.22)**
100	40	1.8	**2.90 (2.02–3.77)**	**2.54 (1.65–3.42)**	**2.20 (1.32–3.08)**
Carrying/lifting	2280				
0	1006	44.1	0	0	0
12.5	691	30.3	**0.81 (0.54–1.08)**	**0.73 (0.46-1.00)**	**0.60 (0.33–0.87)**
25	289	12.7	**1.58 (1.22–1.94)**	**1.44 (1.07–1.82)**	**1.23 (0.85–1.60)**
50	135	5.9	**2.48 (1.98–2.98)**	**2.30 (1.79–2.81)**	**1.86 (1.35–2.37)**
75	91	4.0	**2.42 (1.82–3.01)**	**2.20 (1.60–2.80)**	**1.87 (1.27–2.46)**
100	68	3.0	**3.55 (2.87–4.23)**	**3.33 (2.63–4.02)**	**2.93 (2.23–3.62)**

Model 1: Adjusted for age. Model 2: model 1 + education. Model 3: model 2 + influence at work, emotional demands, and osteoarthritis. Statistically significant differences are marked in bold

**Table 4:** Associations between level of specific ergonomic exposures and differences in low-back pain (LBP) intensity compared to no exposure among *men*, presented as difference in LBP intensity and 95% confidence intervals

% of work time	*N*	%	Model 1	Model 2	Model 3
Standing/walking	1550				
0	21	1.4	0	0	0
12.5	378	24.4	0.60 (− 0.55-1.74)	0.59 (− 0.54-1.72)	0.53 (− 0.58-1.65)
25	361	23.3	0.51 (− 0.64-1.65)	0.52 (− 0.61-1.65)	0.45 (− 0.67-1.56)
50	329	21.2	0.88 (− 0.27-2.03)	0.73 (− 0.40-1.87)	0.53 (− 0.59-1.66)
75	215	13.9	**1.22 (0.06–2.39)**	1.04 (− 0.12-2.20)	0.88 (− 0.26-2.02)
100	246	15.9	**1.23 (0.06–2.39)**	0.97 (− 0.18-2.13)	0.80 (− 0.34-1.95)
Back bent/twisted	1550				
0	492	31.7	0	0	0
12.5	621	40.1	**0.49 (0.19–0.79)**	**0.40 (0.10–0.71)**	0.29 (− 0.01-0.59)
25	190	12.3	**1.26 (0.83–1.68)**	**1.07 (0.62–1.51)**	**0.88 (0.44–1.32)**
50	125	8.1	**1.27 (0.77–1.77)**	**1.01 (0.50–1.52)**	**0.82 (0.32–1.33)**
75	71	4.6	**2.11 (1.48–2.75)**	**1.95 (1.31–2.58)**	**1.78 (1.15–2.79)**
100	51	3.3	**2.62 (1.88–3.35)**	**2.30 (1.56–3.05)**	**2.05 (1.30–2.79)**
Arms elevated	1550				
0	1387	36.0	0	0	0
12.5	1623	42.1	**0.55 (0.25–0.85)**	**0.47 (0.16–0.77)**	**0.35 (0.05–0.66)**
25	466	12.1	**1.39 (0.98–1.80)**	**1.22 (0.80–1.65)**	**1.04 (0.61–1.47)**
50	209	5.4	**1.16 (0.60–1.72)**	**0.94 (0.36–1.53)**	**0.71 (0.13–1.29)**
75	101	2.6	**2.76 (1.94–3.58)**	**2.48 (1.65–3.32)**	**2.32 (1.49–3.14)**
100	67	1.7	**1.55 (0.53–2.56)**	**1.22 (0.20–2.25)**	1.00 (− 0.01-2.02)
Repetitive work	1550				
0	467	30.1	0	0	0
12.5	697	45.0	**0.55 (0.25–0.85)**	**0.43 (0.12–0.73)**	**0.34 (0.04–0.65)**
25	222	14.3	**1.09 (0.59–1.59)**	**0.78 (0.27–1.29)**	**0.67 (0.17–1.18)**
50	97	6.3	**1.01 (0.44–1.59)**	**0.85 (0.26–1.43)**	**0.70 (0.12–1.27)**
75	41	2.7	**1.14 (0.39–1.89)**	**0.91 (0.16–1.66)**	**0.78 (0.04–1.53)**
100	26	1.7	**1.43 (0.75–2.12)**	**1.14 (0.44–1.83)**	**0.91 (0.22–1.61)**
Squatting/kneeling	1550				
0	810	52.3	0	0	0
12.5	425	27.4	**0.52 (0.22–0.82)**	**0.41 (0.10–0.71)**	**0.32 (0.02–0.63)**
25	121	7.8	**1.00 (0.57–1.44)**	**0.83 (0.37–1.28)**	**0.69 (0.24–1.14)**
50	86	5.6	**1.20 (0.62–1.78)**	**1.00 (0.41–1.60)**	**0.79 (0.24–1.14)**
75	49	3.2	**1.05 (0.21–1.88)**	0.81 (− 0.03-1.65)	0.66 (− 0.17-1.50)
100	59	3.8	**1.47 (0.49–2.45)**	**1.04 (0.05–2.03)**	**0.91 (0.22–1.61)**
Pushing/pulling	1550				
0	763	49.2	0	0	0
12.5	467	30.1	**0.58 (0.29–0.87)**	**0.48 (0.18–0.78)**	**0.43 (0.13–0.73)**
25	167	10.8	**1.28 (0.84–1.71)**	**1.06 (0.60–1.52)**	**0.94 (0.48–1.39)**
50	86	5.6	**1.27 (0.62–1.91)**	**1.03 (0.36–1.70)**	**0.86 (0.19–1.52)**
75	39	2.5	**2.38 (1.37–3.38)**	**2.10 (1.08–3.12)**	**2.05 (1.05–3.06)**
100	28	1.8	**2.93 (2.04–3.82)**	**2.56 (1.65–3.47)**	**2.43 (1.52–3.33)**
Carrying/lifting	1550				
0	756	48.8	0	0	0
12.5	505	32.6	**0.49 (0.19–0.79)**	**0.41 (0.10–0.71)**	**0.33 (0.02–0.64)**
25	164	10.6	**0.76 (0.35–1.17)**	**0.59 (0.16–1.03)**	**0.47 (0.04–0.90)**
50	66	4.3	**1.49 (0.99-2.00)**	**1.23 (0.70–1.76)**	**1.07 (0.54–1.60)**
75	26	1.7	**1.53 (0.83–2.23)**	**1.31 (0.58–2.04)**	**1.17 (0.45–1.89)**
100	33	2.1	**2.88 (2.12–3.63)**	**2.62 (1.84–3.39)**	**2.41 (1.64–3.18)**

Model 1: Adjusted for age. Model 2: model 1 + education. Model 3: model 2 + influence at work, emotional demands, and osteoarthritis. Statistically significant differences are marked in bold

#### Neck/shoulder pain (NSP)

While no associations existed between ‘standing/walking’ and NSP, associations existed in most levels of all other ergonomic exposures (Table 5). In the minimally adjusted model (Model 1), an association existed at all levels of all exposures, but when adjusting for education, psychosocial work factors, and osteoarthritis (Model 2–3), some associations vanished. The* p*-value for the trend test for ‘standing/walking’ was < 0.05, while all other combinations showed very strong exposure-response associations (trend test:* p *< 0.0001).

**Table 5:** Associations between level of specific ergonomic exposures and neck/shoulder pain (NSP) intensity compared to workers with no exposure, presented as difference in NSP intensity and 95% confidence intervals

% of work time	*N*	%	Model 1	Model 2	Model 3
Standing/walking	3853				
0	47	1.2	0	0	0
12.5	980	25.4	− 0.66 (− 1.43-0.12)	− 0.62 (− 1.39-0.15)	− 0.53 (− 1.28-0.22)
25	756	19.6	− 0.59 (− 1.37-0.19)	− 0.56 (− 1.33-0.21)	− 0.60 (− 0.35-0.15)
50	742	19.3	− 0.37 (− 1.15-0.41)	− 0.43 (− 1.20-0.34)	− 0.67 (− 1.43-0.08)
75	557	14.5	− 0.19 (− 0.98-0.60)	− 0.29 (− 1.07-0.49)	− 0.54 (− 1.31-0.22)
100	771	20.0	0.31 (− 0.47-1.09)	0.08 (− 0.69-0.85)	− 0.20 (− 0.96-0.55)
Back bent / twisted	3853				
0	1456	37.8	0	0	0
12.5	1316	34.2	**0.64 (0.45–0.84)**	**0.54 (0.34–0.73)**	**0.39 (0.19–0.58)**
25	437	11.3	**1.25 (0.97–1.53)**	**1.07 (0.79–1.35)**	**0.81 (0.53–1.09)**
50	287	7.5	**1.55 (1.23–1.88)**	**1.35 (1.02–1.68)**	**1.06 (0.73–1.39)**
75	185	4.8	**1.90 (1.51–2.30)**	**1.73 (1.33–2.12)**	**1.46 (1.07–1.86)**
100	172	4.5	**2.54 (2.13–2.94)**	**2.23 (1.82–2.65)**	**1.90 (1.48–2.31)**
Arms elevated	3853				
0	1387	36.0	0	0	0
12.5	1623	42.1	**0.54 (0.35–0.73)**	**0.43 (0.24–0.62)**	**0.22 (0.03–0.42)**
25	466	12.1	**1.20 (0.93–1.48)**	**1.00 (0.72–1.28)**	**0.72 (0.43-1.00)**
50	209	5.4	**1.72 (1.34–2.10)**	**1.49 (1.11–1.88)**	**1.12 (0.74–1.51)**
75	101	2.6	**2.25 (1.72–2.77)**	**1.97 (1.43–2.50)**	**1.67 (1.14–2.19)**
100	67	1.7	**2.05 (1.41–2.69)**	**1.70 (1.06–2.34)**	**1.37 (0.74–2.01)**
Repetitive work	3853				
0	2368	61.5	0	0	0
12.5	838	21.8	**0.56 (0.35–0.77)**	**0.45 (0.24–0.66)**	**0.33 (0.13–0.54)**
25	232	6.0	**1.31 (0.96–1.67)**	**1.05 (0.69–1.41)**	**0.93 (0.58–1.28)**
50	153	4.0	**1.23 (0.80–1.66)**	**1.05 (0.61–1.48)**	**0.94 (0.52–1.36)**
75	107	2.8	**1.54 (1.03–2.05)**	**1.31 (0.80–1.82)**	**1.16 (0.66–1.66)**
100	155	4.0	**1.63 (1.21–2.06)**	**1.35 (0.92–1.78)**	**1.26 (0.83–1.68)**
Squatting/kneeling	3853				
0	2056	53.4	0	0	0
12.5	1065	27.6	**0.41 (0.22–0.61)**	**0.27 (0.07–0.46)**	0.09 (− 0.10-0.29)
25	424	11.0	**1.24 (0.96–1.52)**	**1.05 (0.77–1.33)**	**0.74 (0.46–1.02)**
50	175	4.5	**1.35 (0.92–1.76)**	**1.12 (0.71–1.53)**	**0.78 (0.38–1.19)**
75	78	2.0	**0.79 (0.20–1.39)**	0.50 (− 0.10-1.09)	0.29 (-0.29-0.87)
100	55	1.4	**1.94 (1.24–2.65)**	**1.64 (0.94–2.35)**	**1.47 (0.79–2.18)**
Pushing/pulling	3853				
0	2119	55.0	0	0	0
12.5	1075	27.9	**0.36 (0.17–0.56)**	**0.24 (0.04–0.43)**	0.10 (-0.09-0.30)
25	372	9.7	**1.08 (0.79–1.37)**	**0.88 (0.58–1.17)**	**0.62 (0.32–0.91)**
50	147	3.8	**1.85 (1.41–2.28)**	**1.59 (1.15–2.04)**	**1.25 (0.81–1.68)**
75	66	1.7	**2.06 (1.42–2.70)**	**1.71 (1.07–2.35)**	**1.44 (0.81–2.07)**
100	74	1.9	**2.71 (2.10–3.31)**	**2.30 (1.69–2.92)**	**2.10 (1.50–2.71)**
Carrying/lifting	3853				
0	1564	40.6	0	0	0
12.5	1270	33.0	**0.28 (0.09–0.47)**	0.18 (− 0.01-0.38)	0.06 (− 0.13-0.26)
25	502	13.0	**0.75 (0.48–1.01)**	**0.55 (0.28–0.82)**	**0.36 (0.09–0.63)**
50	255	6.6	**1.53 (1.18–1.87)**	**1.26 (0.90–1.62)**	**0.94 (0.58–1.29)**
75	146	3.8	**1.32 (0.87–1.76)**	**1.02 (0.56–1.47)**	**0.75 (0.30–1.20)**
100	116	3.0	**2.68 (2.19–3.18)**	**2.37 (1.87–2.88)**	**2.04 (1.55–2.54)**

Model 1: Adjusted for age and sex. Model 2: model 1 + education. Model 3: model 2 + influence at work, emotional demands, and osteoarthritis. Statistically significant differences are marked in bold

#### Correlations between exposure variables

The Spearman correlations between the seven ergonomic exposure variables ranged from 0.35 to 0.74, indicating moderate to strong correlations (cf. Table 6). The weakest correlation was observed between ‘standing/walking’ and ‘repetitive arm movements’, whereas the strongest correlation was observed between ‘pushing/pulling’ and ‘carrying/lifting’.

**Table 6:** Spearman correlation matrix between the seven single ergonomic exposure variables

Exposure	Standing/walking	Back bent or twisted	Arms lifted	Repetitive arm movements	Squatting/kneeling	Pushing/pulling	Carrying/lifting
Standing/walking	1	0.43	0.55	0.35	0.50	0.52	0.56
Back bent or twisted	0.43	1	0.61	0.44	0.53	0.55	0.55
Arms lifted	0.55	0.61	1	0.50	0.62	0.65	0.68
Repetitive arm movements	0.35	0.44	0.50	1	0.40	0.50	0.51
Squatting/kneeling	0.50	0.53	0.62	0.40	1	0.68	0.66
Pushing/pulling	0.52	0.55	0.65	0.50	0.68	1	0.74
Carrying/lifting	0.56	0.55	0.68	0.51	0.66	0.74	1

### Ergonomic index and musculoskeletal pain

**Fig. 1:**
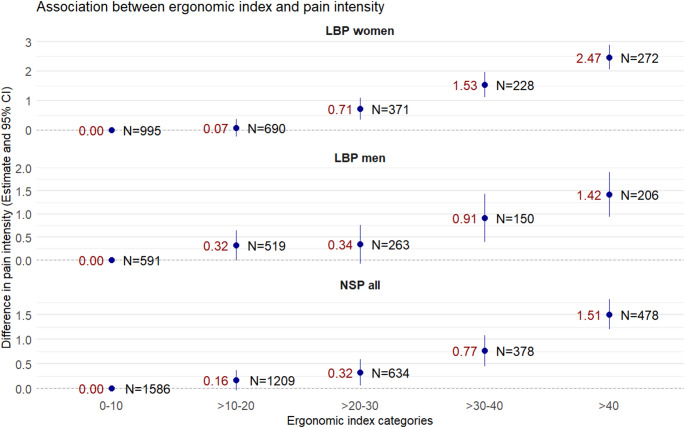
Associations between ergonomic index (0-100) divided in five categorical variables and differences in low-back pain (LBP) intensity for women (upper figure), LBP intensity for men (middle figure), and neck/shoulder pain (NSP) intensity for all workers (lower figure), respectively, compared to workers with an ergonomic index between 0–10 (reference). Analyses for LBP are adjusted for age, education, influence at work, emotional demands, and osteoarthritis, while analyses for NSP are adjusted for age, sex, education, influence at work, emotional demands, and osteoarthritis

Figure 1 shows the associations between ergonomic index with LBP intensity among women, LBP intensity among men, and NSP intensity among all workers (women and men combined), respectively. For all three analyses, clear exposure-response associations existed between higher ergonomic work demands and higher pain intensity (trend tests: *p* < 0.0001).

Compared to the reference group (ergonomic index 0–10), women with an ergonomic index between > 10–20 did not show higher LBP intensity (0.07 points (95% CI − 0.22−0.37,* p*-value = 0.632), while men with an ergonomic index > 10–20 showed a tendency for higher LBP intensity (0.32 points (95% CI − 0.00−0.64, *p*-value = 0.053)). Only women showed higher LBP intensity of 0.71 points (95% CI 0.35–1.08) with ergonomic indexes of > 20−30. Both women and men had higher LBP intensities at ergonomic indexes of > 30–40: 1.53 points (95% CI 1.10–1.97) for women and 0.91 points (95% CI 0.39–1.43) for men. Lastly, women and men with ergonomic indexes > 40 had higher LBP intensity of 2.47 points (95% CI 2.05–2.88) for women and 1.42 points (95% CI 0.93–1.90) for men, respectively.

While no association was observed between ergonomic index between > 10–20 and NSP intensity, workers with ergonomic indexes between > 20–30 had higher NSP intensity of 0.32 points (95% CI 0.06–0.56). Lastly, workers with ergonomic indexes between > 30–40 and > 40 showed higher NSP intensity of 0.77 points (95% CI 0.45–1.08) and 1.51 points (95% CI 1.20–1.71), respectively.

### Sensitivity analyses

Excluding participants with serious illness (Supplementary Table 1, models 3a) or restricting the sample to workers working 20–60 h/week (Supplementary Table 1, models 3b) showed similar estimates as the main analyses (i.e. models 3 in Tables 3, 4 and 5). All analyses were fully adjusted models. Some significances disappeared among men due to wider CIs and low sample sizes in some exposure categories. When including only workers working between 35 and 45 h/week (Supplementary Table 3), most associations for LBP intensity remained statistically significant among women, although at a slightly lower level. For LBP intensity among men, the estimates were also lower and more differences disappeared, seemingly to a random manner due to low sample sizes in the exposure groups.

For NSP, associations and estimates closely mirrored those of the main analyses for workers without serious illness and workers working 20–60 h/week (cf. Supplementary Table 2). For workers working 35–45 h/week showed somewhat lower estimates, where some differences vanished (cf. Supplementary Table 4).

## Discussion

Our study provides the first mapping of ergonomic exposures and their associations with musculoskeletal pain among the general working population in the Faroe Islands. The findings show that Faroese workers experience relatively high levels of musculoskeletal pain, with mean intensities of 2.6 and 3.0 points for LBP and NSP, respectively, on a 0–10 NRS. Most specific ergonomic exposures showed statistically significant exposure-response associations with both LBP and NSP intensity. Importantly, clear exposure-response associations were observed between cumulative ergonomic work demands and pain intensity, with workers exposed to the highest levels of ergonomic demands (ergonomic index > 40) showing substantially higher pain intensities. Comparing ergonomic indexes > 40 to those with low ergonomic exposure (ergonomic index ≤ 10), LBP intensities were 2.47 points higher for women and 1.42 higher points for men, respectively, while NSP intensity were 1.51 points higher among all workers (women and men combined).

The observed exposure-response associations between specific ergonomic exposures and musculoskeletal pain intensity concurs with previous prospective studies and reviews showing that physically demanding work increases the risk of developing musculoskeletal pain (da Costa and Vieira 2010; Andersen et al. 2021a, b; Kuijer et al. 2024). Our findings are particularly important as they show consistent exposure-response associations across multiple ergonomic exposures, with the strongest associations observed for ‘pushing/pulling’ and ‘carrying/lifting’ with LBP intensity, and ‘back bent/twisted’ and ‘pushing/pulling’ with NSP intensity. These results support previous evidence that workers in physically demanding occupations face cumulative exposure to multiple ergonomic risk factors throughout their working day, which may synergistically increase their physical work demands and associated risk (Andersen et al. 2021a, b; Andersen et al. 2021a, b). 

The correlations between the seven ergonomic exposure variables were moderate to strong, with Spearman correlation coefficients ranging from 0.35 to 0.74 (cf. Table 5). This indicates that workers reporting high exposure to one ergonomic factor also tended to report higher exposure to other ergonomic factors. The strongest correlation was observed between pushing/pulling and carrying/lifting, suggesting substantial overlap between these physically demanding work tasks. The moderate-to-strong correlations support the inclusion of both the individual ergonomic exposure variables and the composite ergonomic index. While the individual exposure analyses provide information about specific work-related ergonomic factors, the ergonomic index captures the accumulated or overall ergonomic exposure burden. Thus, the moderate-to-strong correlations between the exposure variables support the interpretation of the ergonomic index as a meaningful summary measure of overall ergonomic workload. Furthermore, because the correlations were below levels that would indicate complete overlap, the exposure-specific analyses remain relevant for identifying which particular ergonomic factors are associated with LBP and NSP. 

The pain intensity levels observed in our study are clinically significant given recent evidence from cohort studies showing dose-response associations between higher musculoskeletal pain intensity and increased risk of long-term sickness absence and disability pension (Skovlund et al. 2023; Bláfoss et al. 2021). These studies found LBP and NSP intensities of 3 and 4, respectively, to increase the risk of long-term sickness absence among the general working population in Denmark (Skovlund et al. 2023) and pain intensities of ≥ 3 in the low back, neck/shoulders, and knees to increase the risk of disability pension (Bláfoss et al. 2021). In the present study, mean LBP and NSP intensities were 2.6 and 3.0, respectively, with those in the highest ergonomic exposure categories reporting pain levels above 4–5 points, suggesting that a considerable proportion of the Faroese workforce may face similar risks. In fact, compared with a recent Danish cohort reporting mean LBP and NSP intensities of 2.4 and 2.7, respectively, on a 0–10 NRS (Andersen et al. 2021a, b), pain levels in the Faroese workforce appear slightly higher. In the present study, women seem to have slightly higher LBP levels than men in the present study, indicating that particularly women may be at increased risk of more serious health outcomes. Although these differences may not be clinically meaningful and populations are not directly comparable, the findings indicate that Faroese workers, particularly female workers, are unlikely to have lower risks of adverse outcomes. Therefore, this may have relevance for future preventive initiatives to maintain the highly active workforce, as the Faroe Islands stand out with the highest labor force participation rate in Europe, with 85% of people aged 15–74 active in the labor force and an 83% participation rate among women, compared to 75% in Iceland, which holds the second‑highest rate among women (Grundfelder et al. 2020; Hagstovan 2025b).

In the present study, FISCO-groups representing physically demanding occupations were smaller than those with predominantly sedentary work (cf. Table 2). Notably, FISCO-group ‘6: Agriculture, forestry, and fishing, excluding assistants’ was the smallest, which might seem inconsistent with the Faroe Islands’ historical profile as a fishing and agricultural society. However, despite the fishing industry’s continued economic importance (> 2000 workers) (Hagstovan 2025a, b), selection bias likely occurred, as workers in physically strenuous occupations (FISCO-groups 6–9) were underrepresented (Hagstovan 2025a). Nonetheless, differences occurred in musculoskeletal pain intensities across FISCO-groups, ranging from 1.84–3.84 points for LBP and 2.34–4.14 points for NSP. A larger sample of high-exposure groups would increase the robustness in the analyses, which may influence the mean pain intensities and associations. However, the present study illustrates a clear pattern that the more physically demanding occupations report higher pain intensities, which concurs with the scientific literature (da Costa and Vieira 2010; Andersen et al. 2021a, b; Kuijer et al. 2024). This trend becomes even more evident in FISCO-11, where refined subgrouping enhances differentiation by ergonomic demands, capturing variation in working conditions more accurately than the original FISCO-code.

Although showing consistent exposure-response associations across ergonomic exposures and models, the estimates generally decreased from model 1 to model 3 (cf. Tables 3 and 4), suggestive of a confounding effect. Adjusting for education reduced most estimates, and further adjustment for psychosocial work factors and osteoarthritis led to additional attenuation. Because educational level is a well-established socioeconomic indicator that is strongly and consistently associated with lifestyle factors as body weight, smoking status, and leisure-time physical activity in Nordic populations (Danish Health Authority 2022), it likely captured part of their confounding influence. These findings suggest that education, and likely the related lifestyle factors, partly explain the association between ergonomic exposures and musculoskeletal pain intensity, consistent with previous research (Danish Health Authority 2022; Hansen et al. 2023; Xu et al. 2024). Despite step-wise attenuation, most associations remained statistically significant, while some reached borderline significance, and some associations disappeared. The further reduction of estimates after adjusting for psychosocial factors and diagnosed osteoarthritis (cf. Tables 3 and 4) aligns with previous research showing influence at work and emotional demands as risk factors for LBP, even after adjusting for physical workload (Clausen et al. 2013). Furthermore, osteoarthritis likely acted as a confounder by affecting both the way the workers cope with their ergonomic exposures and their pain intensity directly. Consequently, the lower estimates in model 3 may be related to both the psychosocial work factors and osteoarthritis. However, post hoc analysis excluding osteoarthritis in model 3 (data not presented) showed only second-decimal changes, indicating minimal impact on the overall findings.

The sex-stratified analyses showed that Faroese female workers had slightly higher LBP intensities compared to male workers, although the differences may not be clinically meaningful (Dworkin et al. 2008). Besides elaborating on the scientific literature that women experience more pain than men (Hartvigsen et al. 2018; Christiansen et al. 2024), the included women in the present study could be exposed to job tasks that the ergonomic exposures may not capture, thereby leading to higher pain intensities. Furthermore, anatomic and/or physiological differences between women and men could potentially influence these findings. However, the lower sample size among male workers in the present study may also explain some of the variation, as the differences were relatively small with women having 0.33 points (95% CI 0.15–0.52) higher LBP intensity than men.

Because the main analyses included workers with serious illnesses (e.g. cancer, myocardial infarction, ischemic stroke) that may influence musculoskeletal pain (Hamood et al. 2018; Schmitz et al. 2024), a sensitivity analysis was conducted to examine their impact (cf. models 3a in Supplementary Table 1). The sensitivity analysis showed that the number of workers with serious illness was low, and estimates were nearly identical with those in model 3 (Tables 3 and 4), indicating minimal influence of serious illness, although some differences disappeared among male workers (cf. Supplementary Table 1, Models 3). Moreover, the weekly working hour responses in this study showed large variations; therefore, two sensitivity analyses addressed this variation by including only workers with weekly working hours between 20 and 60 h/week (cf. Supplementary Table 1) and 35–45 h/week (cf. Supplementary Table 3), as a full-time job according to the Faroese legislation consists of 40 h/week. Consequently, the sensitivity analysis showed that most workers worked between 20 and 60 h/week, and the estimates again mirrored the main analyses (cf. models 3 in Tables 3 and 4 & models 3b in Supplementary Table 1). When comparing the estimates in the main analysis with workers working 35–45 h/week, LBP estimates were slightly lower in the sensitivity analyses among women and men, and some associations disappeared particularly among men (e.g. for squatting/kneeling and pushing/pulling). This was also the case for NSP intensity among all workers (cf. Supplementary Table 4). Collectively, the sensitivity analyses might suggest that workers working around the normal weekly working hours (i.e. 35–45 h/week) may have the necessary capacity required for their job. However, as most of the vanished associations are on the higher exposure levels, it may rather indicate low statistical power in this specific sensitivity analysis. However, the results from the sensitivity analyses excluding workers with serious illness and including workers working 20–60 h/week indicate strong robustness in the main analyses.

### Practical implications

The Faroe Islands presents a unique context for workplace health interventions. Based on our findings, intervention studies should focus on the effect of reducing pushing/pulling and carrying/lifting techniques, which showed the strongest associations with musculoskeletal pain. Other preventions known to prevent and reduce musculoskeletal pain – such as workplace health promotion – may also be considered (Andersen 2024). In fact, the report of the descriptive comparison between the work environment and health in the Faroe Islands and Denmark in this nationwide survey showed that a markedly smaller fraction of the Faroese workforce were offered health promotion initiatives at their workplace (Christiansen et al. 2024). This shows a potential for Faroese workplace to provide health promoting offers for their employees. Focusing on workplace health promotion also elaborates on the recently developed CoWork musculoskeletal health model (Holtermann et al. 2026), which is a paradigm shift from focusing on reducing risks for musculoskeletal *disorders* to an increased focus on improving musculoskeletal *health* through workplace health promotion initiatives (Holtermann et al. 2026). However, before recommending workplace health promotion activities across the working population in the Faroe Islands, the present study’s mapping of the work environment and health remains essential. In the present study, women and men in the highest ergonomic demand category (ergonomic index > 40) had LBP intensity levels of 2.47 points and 1.42 points, respectively, while NSP intensity among all workers remained 1.51 points higher than those with low ergonomic exposures (ergonomic index ≤ 10). For a small economy heavily dependent on physical industries, these pain levels, particularly among female workers, represent both an urgent public health concern and an economic imperative for intervention. The country’s unique capacity for population-level interventions creates conditions for generating robust evidence about workplace health improvement strategies.

### Limitations and strengths

Our study has some limitations. First, the cross-sectional design precludes inferences about the causal association between ergonomic exposures and musculoskeletal pain, as temporal relationships cannot be established. However, the present study elaborates on previous prospective studies and reviews observing ergonomic exposures as risk factors for the development of LBP and long-term sickness absence (da Costa and Vieira 2010; Andersen et al. 2021a, b; Kuijer et al. 2024). Second, all data were collected through self-reports, which may introduce recall bias and subjective interpretation of ergonomic exposures. On the other hand, pain intensity is, per definition, a subjective experience. Workers experiencing higher pain intensities may be more likely to report higher ergonomic exposure, potentially inflating the observed associations. In line, common method variance may be present where, e.g., the mood, health status, and socioeconomic status may influence the answers (Podsakoff et al. 2003). Third, although we adjusted for relevant confounders including age, sex, education, psychosocial work factors, and osteoarthritis, residual confounding from unmeasured variables such as physical fitness, body mass index, lifestyle factors, or genetic predisposition may exist. Fourth, the response rate and potential selection bias should be considered, as workers with musculoskeletal problems may be more motivated to participate in a study about work-related health issues. Moreover, the present study seem to be prone to selection bias as the sample sizes in FISCO-groups 6–9 were lower compared to FISCO-groups 1–5, which does not directly reflect the distribution of the Faroese workforce (Hagstovan 2025a). Lastly, a limitation may be that the same ergonomic exposure variables were examined for both LBP and NSP, although the anatomical relevance of each exposure may differ by pain region. Exposures involving trunk posture, lifting/carrying, pushing/pulling, standing/walking, and squatting/kneeling are likely to be most directly relevant for LBP, whereas arm elevation, repetitive work, pushing/pulling, and carrying/lifting are more directly relevant for NSP. Other exposures, such as standing/walking, may be less directly related to neck/shoulder structures and may instead reflect physically demanding work more generally. Therefore, exposure-specific associations should be interpreted in light of anatomical plausibility, while the ergonomic index should be understood as a measure of overall ergonomic workload.

The study also has strengths. First, this study represents the first mapping of ergonomic exposures and musculoskeletal pain in the Faroese workforce, with approximately 18% of the country’s entire working population included in the main analyses, providing unprecedented population-level knowledge. Second, the large sample size of 3800 workers enabled detection of robust exposure-response associations across multiple ergonomic factors. Third, the approach of examining both specific ergonomic exposures and a combined ergonomic index provides a more complete understanding of cumulative work demands. Fourth, the systematic adjustment for relevant confounders, including psychosocial work factors from the validated Danish Psychosocial Work Environment Questionnaire (Clausen et al. 2019), as well as the sensitivity analyses performed, strengthens the validity of the observed associations. Finally, the unique context of the Faroe Islands as a small, relatively homogeneous society with potential for high intervention adherence makes these findings particularly valuable for informing targeted workplace health initiatives.

### Concluding remarks

The present study found relatively high musculoskeletal pain intensities among the general working population in the Faroe Islands, and clear exposure-response associations of higher levels of specific ergonomic exposures during work and higher LBP and NSP intensities. Furthermore, the study showed clear exposure-response associations between higher overall ergonomic work demands and higher musculoskeletal pain intensity. The present study is the first mapping of the ergonomic working environment among the general working population in the Faroe Islands, which provides crucial knowledge for conducting relevant initiatives to ensure safe and healthy work environments among the Faroese working population. As a small society, the Faroe Islands may contain a large potential to conduct country-wide initiatives with high adherence, which may serve as vital knowledge for larger populations to translate and scale up the initiatives.

## Supplementary Information

Below is the link to the electronic supplementary material.

Supplementary Material 1

## Data Availability

Data is available upon reasonable request. The authors encourage collaboration and use of the data by other researchers. Data are stored on a secure server at the National Research Centre for the Working Environment, and researchers interested in using the data for scientific purposes should contact annikad@setur.fo.
