# Supplementary material for: First evidence on ergonomic exposures and musculoskeletal pain among the Faroese workforce
Source: Int Arch Occup Environ Health. 2026 Jul 6;99(5):33. doi: 10.1007/s00420-026-02221-7 (PMC13337627; doi:10.1007/s00420-026-02221-7)
Supplement: Supplementary file 1 — Supplementary Material 1 [file 420_2026_2221_MOESM1_ESM.docx]

# **SUPPLEMENTARY MATERIAL**

**Supplementary Table 1.** Sensitivity analyses of associations between level of specific ergonomic exposures and differences in LBP intensity compared to no exposure among women and men, presented as difference in LBP intensity and 95% confidence intervals. Analyses are presented as model 3a and 3b for LBP among women (upper table) and LBP among men (lower table), respectively. Model 3a: Workers with serious illness excluded. Model 3b: Only workers working between 20-60 hours/week included.

| % of work time and pain site | N | % | Model 3a | N | % | Model 3b |
| --- | --- | --- | --- | --- | --- | --- |
| **Women - LBP** |  |  | Estimates 95% CI |  |  | Estimates 95% CI |
| Standing/walking | 2244 |  |  | 2173 |  |  |
| 0 | 25 | 1.1 | 0 | 24 | 1.1 | 0 |
| 12.5 | 582 | 25.9 | 0.51 (-0.59-1.61) | 577 | 26.6 | 0.67 (-0.44-1.79) |
| 25 | 375 | 16.7 | 0.64 (-0.48-1.75) | 372 | 17.1 | 0.78 (-0.35-1.91) |
| 50 | 403 | 18.0 | 0.77 (-0.34-1.89) | 386 | 17.8 | 0.87 (-0.26-1.99) |
| 75 | 340 | 15.2 | 0.78 (-0.34-1.90) | 329 | 15.1 | 0.94 (-0.20-2.08) |
| 100 | 519 | 23.1 | **1.60 (0.49-2.71)** | 485 | 22.3 | **1.71 (0.59-2.84)** |
| Back bent / twisted | 2244 |  |  | 2173 |  |  |
| 0 | 935 | 41.7 | 0 | 912 | 42.0 | 0 |
| 12.5 | 673 | 30.0 | **0.50 (0.22-0.77)** | 660 | 30.4 | **0.47 (0.19-0.75)** |
| 25 | 245 | 10.9 | **1.37 (0.97-1.76)** | 233 | 10.7 | **1.45 (1.05-1.85)** |
| 50 | 165 | 7.4 | **1.85 (1.39-2.31)** | 154 | 7.1 | **1.84 (1.37-2.32)** |
| 75 | 112 | 5.0 | **2.41 (1.87-2.96)** | 104 | 4.8 | **2.38 (1.82-2.93)** |
| 100 | 114 | 5.1 | **2.18 (1.63-2.72)** | 110 | 5.1 | **2.15 (1.60-2.70)** |
| Arms elevated | 2244 |  |  | 2173 |  |  |
| 0 | 891 | 39.7 | 0 | 876 | 40.3 | 0 |
| 12.5 | 901 | 40.2 | **0.54 (0.27-0.81)** | 864 | 39.8 | **0.53 (0.26-0.81)** |
| 25 | 247 | 11.0 | **1.27 (0.87-1.68)** | 236 | 10.9 | **1.21 (0.79-1.62)** |
| 50 | 108 | 4.8 | **1.38 (0.82-1.95)** | 100 | 4.6 | **1.37 (0.79-1.95)** |
| 75 | 60 | 2.7 | **1.66 (0.93-2.39)** | 59 | 2.7 | **1.58 (0.85-2.31)** |
| 100 | 37 | 1.7 | **1.36 (0.45-2.27)** | 38 | 1.8 | **1.45 (0.56-2.35)** |
| Repetitive work | 2244 |  |  | 2173 |  |  |
| 0 | 1517 | 67.6 | 0 | 1486 | 68.4 | 0 |
| 12.5 | 401 | 17.9 | **0.42 (0.12-0.73)** | 384 | 17.7 | **0.44 (0.13-0.75)** |
| 25 | 109 | 4.9 | **1.46 (0.92-2.00)** | 102 | 4.7 | **1.35 (0.80-1.90)** |
| 50 | 66 | 2.9 | **1.83 (1.15-2.51)** | 61 | 2.8 | **1.82 (1.12-2.52)** |
| 75 | 55 | 2.5 | **1.37 (0.62-2.12)** | 48 | 2.2 | **1.18 (0.38-1.97)** |
| 100 | 96 | 4.3 | **1.40 (0.82-1.97)** | 92 | 4.2 | **1.36 (0.78-1.95)** |
| Squatting/kneeling | 2244 |  |  | 2173 |  |  |
| 0 | 1251 | 55.8 | 0 | 1225 | 56.4 | 0 |
| 12.5 | 582 | 25.9 | **0.69 (0.41-0.97)** | 553 | 25.5 | **0.69 (0.41-0.98)** |
| 25 | 257 | 11.5 | **1.47 (1.08-1.85)** | 247 | 11.4 | **1.38 (0.99-1.77)** |
| 50 | 91 | 4.1 | **2.06 (1.46-2.65)** | 88 | 4.1 | **2.02 (1.42-2.62)** |
| 75 | 40 | 1.8 | **1.39 (0.53-2.25)** | 37 | 1.7 | **1.47 (0.59-2.36)** |
| 100 | 23 | 1.0 | **2.13 (1.00-3.25)** | 23 | 1.1 | **2.32 (1.20-3.44)** |
| Pushing/pulling | 2244 |  |  | 2173 |  |  |
| 0 | 1321 | 58.9 | 0 | 1292 | 59.5 | 0 |
| 12.5 | 563 | 25.1 | **0.58 (0.31-0.86)** | 541 | 24.9 | **0.57 (0.29-0.85)** |
| 25 | 203 | 9.1 | **1.39 (0.98-1.81)** | 193 | 8.9 | **1.26 (0.84-1.69)** |
| 50 | 78 | 3.5 | **2.04 (1.40-2.68)** | 73 | 3.4 | **1.94 (1.29-2.60)** |
| 75 | 42 | 1.9 | **2.44 (1.59-3.28)** | 39 | 1.8 | **2.23 (1.36-3.11)** |
| 100 | 37 | 1.7 | **2.08 (1.18-2.99)** | 35 | 1.6 | **2.13 (1.20-3.05)** |
| Carrying/lifting | 2244 |  |  | 2173 |  |  |
| 0 | 987 | 43.98 | 0 | 972 | 44.7 | 0 |
| 12.5 | 683 | 30.44 | **0.62 (0.35-0.90)** | 655 | 30.1 | **0.60 (0.33-0.87)** |
| 25 | 288 | 12.83 | **1.23 (0.86-1.61)** | 273 | 12.6 | **1.20 (0.82-1.58)** |
| 50 | 130 | 5.79 | **1.89 (1.37-2.41)** | 128 | 5.9 | **1.86 (1.34-2.38)** |
| 75 | 90 | 4.01 | **1.85 (1.25-2.45)** | 80 | 3.7 | **1.87 (1.24-2.50)** |
| 100 | 66 | 2.9 | **2.90 (2.20-3.59)** | 65 | 3.0 | **2.94 (2.24-3.64)** |
| Table 1 continued |  |  |  |  |  |  |
| % of work time and pain site | N | % | **Model 3a** | N | % | **Model 3b** |
| **Men - LBP** |  |  | **Estimates 95% CI** |  |  | **Estimates 95% CI** |
| Standing/walking | 1482 |  |  | 1329 |  |  |
| 0 | 21 | 1.4 | 0 | 18 | 1.4 | 0 |
| 12.5 | 362 | 24.4 | 0.50 (-0.62-1.61) | 341 | 25.7 | 0.75 (-0.46-1.95) |
| 25 | 340 | 22.9 | 0.44 (-0.67-1.56) | 324 | 24.4 | 0.71 (-0.50-1.92) |
| 50 | 311 | 21.0 | 0.51 (-0.61-1.64) | 256 | 19.3 | 0.79 (-0.43-2.01) |
| 75 | 209 | 14.1 | 0.81 (-0.33-1.95) | 183 | 13.8 | 1.02 (-0.22-2.26) |
| 100 | 239 | 16.1 | 0.72 (-0.42-1.86) | 207 | 15.6 | 1.09 (-0.15-2.33) |
| Back bent / twisted | 1482 |  |  | 1329 |  |  |
| 0 | 479 | 32.3 | 0 | 441 | 33.2 | 0 |
| 12.5 | 588 | 39.7 | 0.27 (-0.04-0.57) | 530 | 39.9 | 0.27 (-0.05-0.60) |
| 25 | 182 | 12.3 | **0.85 (0.40-1.30)** | 166 | 12.5 | **0.82 (0.35-1.30)** |
| 50 | 115 | 7.8 | **0.95 (0.43-1.48)** | 97 | 7.3 | **0.83 (0.26-1.40)** |
| 75 | 68 | 4.6 | **1.85 (1.21-2.49)** | 57 | 4.3 | **1.74 (1.04-2.44)** |
| 100 | 50 | 3.4 | **2.13 (1.39-2.88)** | 38 | 2.9 | **2.15 (1.30-3.00)** |
| Arms elevated | 1482 |  |  | 1329 |  |  |
| 0 | 447 | 30.2 | 0 | 418 | 31.5 | 0 |
| 12.5 | 662 | 44.7 | **0.34 (0.03-0.65)** | 587 | 44.2 | 0.31 (-0.01-0.64) |
| 25 | 216 | 14.6 | **1.00 (0.57-1.43)** | 193 | 14.5 | **0.90 (0.44-1.37)** |
| 50 | 90 | 6.1 | **0.69 (0.09-1.29)** | 82 | 6.2 | **0.72 (0.08-1.35)** |
| 75 | 41 | 2.8 | **2.27 (1.45-3.09)** | 31 | 2.3 | **2.00 (1.06-2.95)** |
| 100 | 26 | 1.8 | 0.93 (-0.08-1.95) | 18 | 1.4 | 0.49 (-0.73-1.71) |
| Repetitive work | 1482 |  |  | 1329 |  |  |
| 0 | 779 | 52.6 | 0 | 715 | 53.8 | 0 |
| 12.5 | 403 | 27.2 | **0.32 (0.01-0.63)** | 351 | 26.4 | 0.29 (-0.04-0.62) |
| 25 | 119 | 8.0 | **0.71 (0.20-1.21)** | 101 | 7.6 | **0.69 (0.14-1.24)** |
| 50 | 74 | 5.0 | **0.83 (0.21-1.44)** | 76 | 5.7 | 0.49 (-0.13-1.10) |
| 75 | 48 | 3.2 | **0.79 (0.04-1.53)** | 43 | 3.2 | 0.78 (-0.02-1.57) |
| 100 | 59 | 4.0 | **0.89 (0.20-1.58)** | 43 | 3.2 | **0.83 (0.02-1.64)** |
| Squatting/kneeling | 1482 |  |  | 1329 |  |  |
| 0 | 726 | 49.0 | 0 | 679 | 51.1 | 0 |
| 12.5 | 449 | 30.3 | 0.29 (-0.02-0.60) | 382 | 28.7 | 0.28 (-0.05-0.61) |
| 25 | 156 | 10.5 | **0.63 (0.17-1.09)** | 146 | 11.0 | **0.66 (0.18-1.15)** |
| 50 | 84 | 5.7 | **0.79 (0.20-1.39)** | 67 | 5.0 | **0.76 (0.09-1.43)** |
| 75 | 39 | 2.6 | 0.63 (-0.21-1.46) | 36 | 2.7 | 0.57 (-0.30-1.44) |
| 100 | 28 | 1.9 | 0.89 (-0.09-1.87) | 19 | 1.4 | 0.53 (-0.66-1.72) |
| Pushing/pulling | 1482 |  |  | 1329 |  |  |
| 0 | 720 | 48.6 | 0 | 681 | 51.2 | 0 |
| 12.5 | 487 | 32.9 | **0.41 (0.11-0.72)** | 415 | 31.2 | **0.41 (0.09-0.74)** |
| 25 | 157 | 10.6 | **0.92 (0.46-1.39)** | 138 | 10.4 | **0.83 (0.34-1.33)** |
| 50 | 59 | 4.0 | **0.93 (0.23-1.62)** | 50 | 3.8 | **0.80 (0.04-1.56)** |
| 75 | 26 | 1.8 | **2.03 (1.03-3.03)** | 19 | 1.4 | **2.27 (1.11-3.44)** |
| 100 | 33 | 2.2 | **2.40 (1.49-3.31)** | 26 | 2.0 | **2.36 (1.33-3.39)** |
| Carrying/lifting | 1482 |  |  | 1329 |  |  |
| 0 | 517 | 34.9 | 0 | 489 | 36.8 | 0 |
| 12.5 | 545 | 36.8 | **0.36 (0.05-0.67)** | 470 | 35.4 | **0.38 (0.05-0.71)** |
| 25 | 198 | 13.4 | 0.41 (-0.03-0.85) | 179 | 13.5 | 0.39 (-0.08-0.85) |
| 50 | 115 | 7.8 | **1.15 (0.61-1.69)** | 102 | 7.7 | **1.30 (0.72-1.88)** |
| 75 | 58 | 3.9 | **1.11 (0.38-1.83)** | 50 | 3.8 | **1.10 (0.32-1.88)** |
| 100 | 49 | 3.3 | **2.37 (1.60-3.15)** | 39 | 2.9 | **2.42 (1.56-3.29)** |

Both models are adjusted for age, education, influence at work, emotional demands, and osteoarthritis. Statistically significant differences are marked in **bold**.

**Supplementary Table 2.** Sensitivity analyses of associations between level of specific ergonomic exposures and differences in NSP intensity compared to no exposure across women and men, presented as difference in NSP intensity and 95% confidence intervals. Analyses are presented as model 3a and 3b. Model 3a: Workers with serious illness excluded. Model 3b: Only workers working between 20-60 hours/week included.

| % of work time and pain site | N | % | Model 3a | N | % | Model 3b |
| --- | --- | --- | --- | --- | --- | --- |
| **NSP** |  |  |  |  |  |  |
| Standing/walking | 3750 |  |  | 3524 |  |  |
| 0 | 47 | 1.3 | 0 | 43 | 1.2 | 0 |
| 12.5 | 952 | 25.4 | -0.54 (-1.29-0.21) | 926 | 26.3 | -0.51 (-1.29-0.27) |
| 25 | 727 | 19.4 | -0.62 (-1.37-0.14) | 707 | 20.1 | -0.54 (-1.33-0.24) |
| 50 | 719 | 19.2 | -0.66 (-1.41-0.10) | 647 | 18.4 | -0.65 (-1.44-0.13) |
| 75 | 548 | 14.6 | -0.55 (-1.31-0.22) | 512 | 14.5 | -0.52 (-1.31-0.28) |
| 100 | 757 | 20.2 | -0.20 (-0.96-0.56) | 689 | 19.6 | -0.15 (-0.94-0.64) |
| Back bent / twisted | 3750 |  |  | 3524 |  |  |
| 0 | 1429 | 38.1 | 0 | 1368 | 38.8 | 0 |
| 12.5 | 1270 | 33.9 | **0.40 (0.21-0.60)** | 1199 | 34.0 | **0.38 (0.18-0.58)** |
| 25 | 427 | 11.4 | **0.83 (0.54-1.11)** | 398 | 11.3 | **0.80 (0.50-1.09)** |
| 50 | 277 | 7.4 | **1.11 (0.78-1.45)** | 248 | 7.0 | **1.02 (0.67-1.38)** |
| 75 | 182 | 4.9 | **1.46 (1.06-1.86)** | 162 | 4.6 | **1.35 (0.93-1.77)** |
| 100 | 165 | 4.4 | **1.93 (1.51-2.35)** | 149 | 4.2 | **1.88 (1.45-2.32)** |
| Arms elevated | 3750 |  |  | 3524 |  |  |
| 0 | 1351 | 36.0 | 0 | 1308 | 37.1 | 0 |
| 12.5 | 1578 | 42.1 | **0.22 (0.03-0.41)** | 1463 | 41.5 | **0.21 (0.01-0.41)** |
| 25 | 458 | 12.2 | **0.73 (0.45-1.02)** | 425 | 12.1 | **0.65 (0.35-0.94)** |
| 50 | 200 | 5.3 | **1.14 (0.75-1.53)** | 183 | 5.2 | **1.34 (0.73-1.54)** |
| 75 | 100 | 2.7 | **1.71 (1.19-2.24)** | 89 | 2.5 | **1.58 (1.03-2.14)** |
| 100 | 63 | 1.7 | **1.26 (0.61-1.91)** | 56 | 1.6 | **1.14 (0.45-1.83)** |
| Repetitive work | 3750 |  |  | 3524 |  |  |
| 0 | 2314 | 61.7 | 0 | 2219 | 63.0 | 0 |
| 12.5 | 811 | 21.6 | **0.33 (0.12-0.54)** | 741 | 21.0 | **0.27 (0.05-0.48)** |
| 25 | 228 | 6.1 | **0.97 (0.62-1.33)** | 201 | 5.7 | **0.89 (0.51-1.26)** |
| 50 | 139 | 3.7 | **1.05 (0.61-1.49)** | 136 | 3.9 | **0.90 (0.46-1.35)** |
| 75 | 106 | 2.8 | **1.18 (0.68-1.68)** | 94 | 2.7 | **1.06 (0.53-1.59)** |
| 100 | 152 | 4.1 | **1.24 (0.81-1.67)** | 133 | 3.8 | **1.18 (0.72-1.63)** |
| Squatting/kneeling | 3750 |  |  | 3524 |  |  |
| 0 | 1997 | 53.3 | 0 | 1924 | 54.6 | 0 |
| 12.5 | 1041 | 27.8 | 0.10 (-0.10-0.30) | 944 | 26.8 | 0.09 (-0.12-0.30) |
| 25 | 410 | 10.9 | **0.78 (0.50-1.07)** | 389 | 11.0 | **0.65 (0.36-0.94)** |
| 50 | 172 | 4.6 | **0.80 (0.39-1.21)** | 152 | 4.3 | **0.71 (0.28-1.15)** |
| 75 | 78 | 2.1 | 0.30 (-0.28-0.88) | 72 | 2.0 | 0.26 (-0.35-0.86) |
| 100 | 52 | 1.4 | **1.41 (0.71-2.12)** | 43 | 1.2 | **1.14 (0.36-1.91)** |
| Pushing/pulling | 3750 |  |  | 3524 |  |  |
| 0 | 2063 | 55.0 | 0 | 1994 | 56.6 | 0 |
| 12.5 | 1050 | 28.0 | 0.13 (-0.07-0.33) | 958 | 27.2 | 0.08 (-0.12-0.28) |
| 25 | 364 | 9.7 | **0.65 (0.35-0.94)** | 333 | 9.5 | **0.49 (0.18-0.80)** |
| 50 | 137 | 3.7 | **1.41 (0.95-1.86)** | 122 | 3.5 | **1.11 (0.64-1.59)** |
| 75 | 65 | 1.7 | **1.43 (0.79-2.06)** | 55 | 1.6 | **1.28 (0.59-1.97)** |
| 100 | 71 | 1.9 | **2.10 (1.48-2.71)** | 62 | 1.8 | **2.11 (1.45-2.77)** |
| Carrying/lifting | 3750 |  |  | 3524 |  |  |
| 0 | 1520 | 40.5 | 0 | 1479 | 42.0 | 0 |
| 12.5 | 1240 | 33.1 | 0.09 (-0.11-0.28) | 1137 | 32.3 | 0.05 (-0.15-0.25) |
| 25 | 490 | 13.1 | **0.38 (0.11-0.65)** | 453 | 12.9 | **0.34 (0.06-0.62)** |
| 50 | 242 | 6.5 | **1.03 (0.67-1.40)** | 226 | 6.4 | **0.94 (0.56-1.31)** |
| 75 | 144 | 3.8 | **0.75 (0.30-1.20)** | 126 | 3.6 | **0.63 (0.15-1.11)** |
| 100 | 114 | 3.0 | **2.05 (1.55-2.56)** | 103 | 2.9 | **2.09 (1.56-2.61)** |

Both models are adjusted for age, sex, education, influence at work, emotional demands, and osteoarthritis. Statistically significant differences are marked in **bold**.

**Supplementary Table 3.** Sensitivity analyses of associations between level of specific ergonomic exposures and differences in LBP intensity compared to no exposure across women and men, presented as difference in LBP intensity and 95% confidence intervals. Analyses are presented as model 3a and 3b. Model 3a: Women working 35-45 hours/week included. Model 3b: Men working 35-45 hours/week included.

| % of work time and pain site | N | % | Model 3a | N | % | Model 3b |
| --- | --- | --- | --- | --- | --- | --- |
| **LBP** |  |  | Estimates 95% CI |  |  | Estimates 95% CI |
| Standing/walking | 1285 |  |  | 868 |  |  |
| 0 | 22 | 1.7 | 0 | 13 | 1.5 | 0 |
| 12.5 | 414 | 32.2 | 0.77 (-0.38-1.93) | 252 | 29.0 | 0.67 (-0.66-2.00) |
| 25 | 233 | 18.1 | 0.91 (-0.27-2.09) | 206 | 23.7 | 0.79 (-0.55-2.12) |
| 50 | 226 | 17.6 | 0.97 (-0.21-2.16) | 163 | 18.8 | 0.92 (-0.44-2.27) |
| 75 | 171 | 13.3 | 1.06 (-0.15-2.26) | 105 | 12.1 | 0.50 (-0.90-1.89) |
| 100 | 219 | 17.0 | **1.55 (0.36-2.74)** | 129 | 14.9 | 0.96 (-0.42-2.35) |
| Back bent / twisted | 1285 |  |  | 868 |  |  |
| 0 | 600 | 46.7 | 0 | 312 | 35.9 | 0 |
| 12.5 | 389 | 30.3 | **0.44 (0.09-0.79)** | 338 | 38.9 | 0.21 (-0.16-0.59) |
| 25 | 115 | 9.0 | **1.88 (1.32-2.43)** | 106 | 12.2 | **0.75 (0.18-1.31)** |
| 50 | 80 | 6.2 | **1.59 (0.96-2.23)** | 59 | 6.8 | **0.82 (0.13-1.50)** |
| 75 | 48 | 3.7 | **1.92 (1.13-2.72)** | 30 | 3.5 | **1.64 (0.74-2.54)** |
| 100 | 53 | 4.1 | **1.47 (0.71-2.24)** | 23 | 2.7 | **1.50 (0.47-2.52)** |
| Arms elevated | 1285 |  |  | 868 |  |  |
| 0 | 599 | 46.6 | 0 | 307 | 35.4 | 0 |
| 12.5 | 478 | 37.2 | **0.58 (0.23-0.92)** | 377 | 43.4 | **0.43 (0.07-0.80)** |
| 25 | 114 | 8.9 | **1.05 (0.48-1.61)** | 100 | 11.5 | **0.72 (0.15-1.29)** |
| 50 | 42 | 3.3 | 0.54 (-0.33-1.41) | 53 | 6.1 | 0.60 (-0.15-1.35) |
| 75 | 31 | 2.4 | **1.68 (0.68-2.67)** | 19 | 2.2 | **1.96 (0.82-3.09)** |
| 100 | 21 | 1.6 | 0.58 (-0.60-1.76) | 12 | 1.4 | -0.20 (-1.61-1.21) |
| Repetitive work | 1285 |  |  | 868 |  |  |
| 0 | 896 | 69.73 | 0 | 495 | 57.0 | 0 |
| 12.5 | 218 | 16.96 | 0.29 (-0.11-0.69) | 214 | 24.7 | 0.22 (-0.17-0.62) |
| 25 | 60 | 4.67 | **1.34 (0.63-2.06)** | 62 | 7.1 | **0.95 (0.30-1.60)** |
| 50 | 30 | 2.33 | **1.97 (0.98-2.97)** | 55 | 6.3 | 0.30 (-0.39-0.98) |
| 75 | 26 | 2.02 | **1.47 (0.40-2.53)** | 22 | 2.5 | **1.38 (0.35-2.41)** |
| 100 | 55 | 4.28 | **1.06 (0.31-1.81)** | 20 | 2.3 | -0.06 (-1.15-1.03) |
| Squatting/kneeling | 1285 |  |  | 868 |  |  |
| 0 | 804 | 62.57 | 0 | 467 | 53.8 | 0 |
| 12.5 | 299 | 23.27 | **0.66 (0.29-1.04)** | 241 | 27.8 | 0.23 (-0.16-0.62) |
| 25 | 116 | 9.03 | **1.44 (0.89-1.98)** | 87 | 10.0 | **0.63 (0.05-1.21)** |
| 50 | 34 | 2.65 | **1.69 (0.76-2.61)** | 40 | 4.6 | 0.65 (-0.17-1.46) |
| 75 | 19 | 1.48 | **1.40 (0.18-2.62)** | 24 | 2.8 | 0.78 (-0.23-1.79) |
| 100 | 13 | 1.01 | **2.17 (0.71-3.63)** | 9 | 1.0 | 0.23 (-1.37-1.84) |
| Pushing/pulling | 1285 |  |  | 868 |  |  |
| 0 | 841 | 65.45 | 0 | 476 | 54.8 | 0 |
| 12.5 | 281 | 21.87 | **0.55 (0.18-0.92)** | 260 | 30.0 | 0.28 (-0.11-0.66) |
| 25 | 87 | 6.77 | **0.87 (0.26-1.48)** | 80 | 9.2 | **0.94 (0.33-1.54)** |
| 50 | 39 | 3.04 | **1.49 (0.61-2.37)** | 29 | 3.3 | 0.00 (-0.94-0.94) |
| 75 | 23 | 1.79 | **2.20 (1.07-3.33)** | 12 | 1.4 | **2.29 (0.91-3.67)** |
| 100 | 14 | 1.09 | **2.72 (1.28-4.16)** | 11 | 1.3 | 0.79 (-0.68-2.26) |
| Carrying/lifting | 1285 |  |  | 868 |  |  |
| 0 | 641 | 49.88 | 0 | 347 | 40.0 | 0 |
| 12.5 | 364 | 28.33 | **0.75 (0.40-1.10)** | 302 | 34.8 | 0.37 (-0.01-0.74) |
| 25 | 148 | 11.52 | **0.89 (0.39-1.39)** | 107 | 12.3 | 0.47 (-0.10-1.03) |
| 50 | 61 | 4.75 | **1.39 (0.66-2.12)** | 61 | 7.0 | **1.16 (0.45-1.88)** |
| 75 | 39 | 3.04 | **2.30 (1.41-3.19)** | 32 | 3.7 | **1.06 (0.14-1.98)** |
| 100 | 32 | 2.49 | **2.67 (1.68-3.65)** | 19 | 2.2 | **1.89 (0.75-3.04)** |

Both models are adjusted for age, education, influence at work, emotional demands, and osteoarthritis. Statistically significant differences are marked in **bold**.

**Supplementary Table 4.** Sensitivity analyses of associations between level of specific ergonomic exposures and differences in NSP intensity compared to no exposure, presented as difference in NSP intensity and 95% confidence intervals. The analysis included women and men working 35-45 hours/week.

| % of work time and pain site | N | % | Model 3 |
| --- | --- | --- | --- |
| **NSP** |  |  | Estimates 95% CI |
| Standing/walking | 2167 |  |  |
| 0 | 35 | 1.6 | 0 |
| 12.5 | 671 | 31.0 | -0.55 (-1.40-0.30) |
| 25 | 449 | 20.7 | -0.51 (-1.37-0.35) |
| 50 | 390 | 18.0 | -0.65 (-1.52-0.22) |
| 75 | 275 | 12.7 | -0.76 (-1.65-0.12) |
| 100 | 347 | 16.0 | -0.32 (-1.20-0.56) |
| Back bent / twisted | 2167 |  |  |
| 0 | 917 | 42.3 | 0 |
| 12.5 | 738 | 34.1 | **0.36 (0.11-0.61)** |
| 25 | 221 | 10.2 | **0.87 (0.49-1.26)** |
| 50 | 134 | 6.2 | **0.88 (0.42-1.35)** |
| 75 | 80 | 3.7 | **0.96 (0.38-1.54)** |
| 100 | 77 | 3.6 | **1.54 (0.95-2.13)** |
| Arms elevated | 2167 |  |  |
| 0 | 911 | 42.0 | 0 |
| 12.5 | 865 | 39.9 | 0.18 (-0.06-0.43) |
| 25 | 215 | 9.9 | **0.56 (0.17-0.95)** |
| 50 | 95 | 4.48 | **0.70 (0.14-1.25)** |
| 75 | 48 | 2.2 | **1.13 (0.38-1.87)** |
| 100 | 33 | 1.5 | 0.39 (-0.49-1.27) |
| Repetitive work | 2167 |  |  |
| 0 | 1399 | 64.6 | 0 |
| 12.5 | 437 | 20.2 | 0.20 (-0.07-0.48) |
| 25 | 121 | 5.6 | **0.97 (0.49-1.44)** |
| 50 | 84 | 3.9 | **0.78 (0.21-1.34)** |
| 75 | 52 | 2.4 | **1.16 (0.46-1.86)** |
| 100 | 74 | 3.4 | **0.94 (0.34-1.54)** |
| Squatting/kneeling | 2167 |  |  |
| 0 | 1279 | 59.0 | 0 |
| 12.5 | 547 | 25.2 | 0.04 (-0.22-0.30) |
| 25 | 205 | 9.5 | **0.49 (0.10-0.88)** |
| 50 | 72 | 3.3 | 0.32 (-0.30-0.93) |
| 75 | 42 | 1.9 | -0.07 (-0.84-0.71) |
| 100 | 22 | 1.0 | **1.16 (0.10-2.22)** |
| Pushing/pulling | 2167 |  |  |
| 0 | 1328 | 61.3 | 0 |
| 12.5 | 542 | 25.0 | 0.00 (-0.26-0.26) |
| 25 | 169 | 7.8 | 0.40 (-0.02-0.82) |
| 50 | 68 | 3.1 | **0.87 (0.24-1.49)** |
| 75 | 34 | 1.6 | **1.00 (0.14-1.86)** |
| 100 | 26 | 1.2 | **2.06 (1.06-3.05)** |
| Carrying/lifting | 2167 |  |  |
| 0 | 999 | 46.1 | 0 |
| 12.5 | 673 | 31.1 | -0.05 (-0.30-0.20) |
| 25 | 255 | 11.8 | 0.10 (-0.26-0.47) |
| 50 | 121 | 5.6 | **0.86 (0.36-1.35)** |
| 75 | 69 | 3.2 | 0.42 (-0.21-1.05) |
| 100 | 50 | 2.3 | **2.28 (1.54-3.01)** |

The model is adjusted for age, sex, education, influence at work, emotional demands, and osteoarthritis. Statistically significant differences are marked in **bold**.

**Supplementary Table 5.** Associations between level of specific ergonomic exposures and differences in LBP intensity compared to no exposure among all workers, presented as difference in LBP intensity and 95% confidence intervals.

| **% of work time** | **N** | **%** | **Model 1** | **Model 2** | **Model 3** |
| --- | --- | --- | --- | --- | --- |
| Standing/walking | 3830 |  |  |  |  |
| 0 | 46 | 1.20 | 0 | 0 | 0 |
| 12.5 | 972 | 25.4 | 0.37 (-0.44-1.19) | 0.42 (-0.39-1.23) | 0.50 (-0.29-1.29) |
| 25 | 743 | 19.4 | 0.55 (-0.27-1.37) | 0.59 (-0.22-1.41) | 0.55 (-0.24-1.35) |
| 50 | 737 | 19.2 | **0.95 (0.13-1.76)** | **0.90 (0.08-1.71)** | 0.65 (-0.14-1.45) |
| 75 | 559 | 14.6 | **1.17 (0.34-1.99)** | **1.11 (0.29-1.93)** | **0.81 (0.01-1.62)** |
| 100 | 773 | 20.2 | **1.82 (1.01-2.64)** | **1.65 (0.83-2.46)** | **1.33 (0.53-2.13)** |
| Back bent / twisted | 3830 |  |  |  |  |
| 0 | 1442 | 37.7 | 0 | 0 | 0 |
| 12.5 | 1306 | 34.1 | **0.68 (0.48-0.88)** | **0.61 (0.40-0.81)** | **0.44 (0.24-0.64)** |
| 25 | 437 | 11.4 | **1.59 (1.30-1.87)** | **1.45 (1.15-1.74)** | **1.16 (0.86-1.45)** |
| 50 | 290 | 7.6 | **1.91 (1.58-2.25)** | **1.75 (1.41-2.09)** | **1.44 (1.10-1.79)** |
| 75 | 184 | 4.8 | **2.62 (2.21-3.03)** | **2.49 (2.08-2.90)** | **2.20 (1.79-2.61)** |
| 100 | 171 | 4.5 | **2.75 (2.33-3.18)** | **2.50 (2.07-2.93)** | **2.13 (1.70-2.56)** |
| Arms elevated | 3830 |  |  |  |  |
| 0 | 1374 | 35.9 | 0 | 0 | 0 |
| 12.5 | 1609 | 42.0 | **0.82 (0.63-1.02)** | **0.74 (0.54-0.94)** | **0.51 (0.31-0.71)** |
| 25 | 471 | 12.3 | **1.69 (1.40-1.98)** | **1.53 (1.23-1.82)** | **1.20 (0.91-1.50)** |
| 50 | 207 | 5.4 | **1.68 (1.28-2.08)** | **1.49 (1.09-1.90)** | **1.08 (0.68-1.48)** |
| 75 | 102 | 2.7 | **2.47 (1.92-3.02)** | **2.24 (1.68-2.79)** | **1.90 (1.35-2.44)** |
| 100 | 67 | 1.8 | **2.01 (1.34-2.67)** | **1.72 (1.04-2.39)** | **1.31 (0.64-1.97)** |
| Repetitive work | 3830 |  |  |  |  |
| 0 | 2351 | 61.4 | 0 | 0 | 0 |
| 12.5 | 831 | 21.7 | **0.65 (0.43-0.87)** | **0.55 (0.33-0.77)** | **0.41 (0.20-0.63)** |
| 25 | 232 | 6.1 | **1.46 (1.09-1.84)** | **1.23 (0.85-1.60)** | **1.05 (0.69-1.42)** |
| 50 | 154 | 4.0 | **1.54 (1.09-1.99)** | **1.37 (0.92-1.82)** | **1.22 (0.77-1.66)** |
| 75 | 104 | 2.7 | **1.51 (0.97-2.05)** | **1.27 (0.72-1.81)** | **1.08 (0.55-1.61)** |
| 100 | 158 | 4.1 | **1.67 (1.23-2.11)** | **1.40 (0.95-1.85)** | **1.22 (0.77-1.66)** |
| Squatting/kneeling | 3830 |  |  |  |  |
| 0 | 2036 | 53.2 | 0 | 0 | 0 |
| 12.5 | 1056 | 27.6 | **0.82 (0.62-1.03)** | **0.72 (0.51-0.93)** | **0.54 (0.33-0.74)** |
| 25 | 427 | 11.2 | **1.64 (1.35-1.93)** | **1.50 (1.21-1.80)** | **1.18 (0.89-1.47)** |
| 50 | 178 | 4.7 | **1.97 (1.55-2.39)** | **1.81 (1.39-2.23)** | **1.45 (1.03-1.87)** |
| 75 | 79 | 2.1 | **1.52 (0.91-2.13)** | **1.31 (0.69-1.92)** | **1.07 (0.47-1.67)** |
| 100 | 54 | 1.4 | **2.10 (1.36-2.84)** | **1.86 (1.12-2.60)** | **1.62 (0.89-2.35)** |
| Pushing/pulling | 3830 |  |  |  |  |
| 0 | 2098 | 54.8 | 0 | 0 | 0 |
| 12.5 | 1075 | 28.1 | **0.76 (0.57-0.96)** | **0.68 (0.47-0.88)** | **0.52 (0.32-0.73)** |
| 25 | 368 | 9.6 | **1.62 (1.32-1.92)** | **1.47 (1.17-1.78)** | **1.20 (0.89-1.51)** |
| 50 | 147 | 3.8 | **2.07 (1.62-2.53)** | **1.89 (1.43-2.35)** | **1.53 (1.07-1.98)** |
| 75 | 69 | 1.8 | **2.79 (2.14-3.44)** | **2.53 (1.88-3.18)** | **2.23 (1.59-2.87)** |
| 100 | 73 | 1.9 | **2.91 (2.28-3.55)** | **2.59 (1.95-3.23)** | **2.31 (1.67-2.94)** |
| Carrying/lifting | 3830 |  |  |  |  |
| 0 | 1548 | 40.4 | 0 | 0 | 0 |
| 12.5 | 1258 | 32.9 | **0.70 (0.50-0.90)** | **0.64 (0.44-0.85)** | **0.52 (0.31-0.72)** |
| 25 | 499 | 13.0 | **1.27 (1.00-1.54)** | **1.17 (0.89-1.45)** | **0.95 (0.67-1.23)** |
| 50 | 258 | 6.7 | **2.05 (1.69-2.41)** | **1.89 (1.52-2.26)** | **1.54 (1.17-1.91)** |
| 75 | 150 | 3.9 | **2.04 (1.58-2.49)** | **1.85 (1.39-2.32)** | **1.58 (1.12-2.03)** |
| 100 | 117 | 3.1 | **3.27 (2.76-3.78)** | **3.08 (2.56-3.60)** | **2.72 (2.20-3.23)** |

Model 1: Adjusted for age and sex. Model 2: model 1 + education. Model 3: model 2 + influence at work, emotional demands, and osteoarthritis. Statistically significant differences are marked in **bold**.
